# Environmental variation shapes genetic variation in *Bouteloua gracilis*: Implications for restoration management of natural populations and cultivated varieties in the southwestern United States

**DOI:** 10.1002/ece3.4767

**Published:** 2018-12-26

**Authors:** Katrina L. Tso, Gerard J. Allan

**Affiliations:** ^1^ Department of Biological Sciences, Environmental Genetics & Genomics Facility Northern Arizona University Flagstaff Arizona

**Keywords:** blue grama, *Bouteloua gracilis*, cultivars, population genetics, restoration, seed source

## Abstract

With the increasing frequency of large‐scale restoration efforts, the need to understand the adaptive genetic structure of natural plant populations and their relation to heavily utilized cultivars is critical. *Bouteloua gracilis* (blue grama) is a wind‐dispersed, perennial grass consisting of several cytotypes (2*n* = 2×–6×) with a widespread distribution in western North America. The species is locally dominant and used regularly in restoration treatments. Using amplified fragment length polymorphism (AFLP) and cpDNA analyses, we assessed the genetic variability and adaptive genetic structure of blue grama within and among 44 sampling sites that are representative of the species’ environmental and habitat diversity in the southwestern United States. Five cultivars were also included to investigate genetic diversity and differentiation in natural versus cultivated populations. Three main findings resulted from this study: (a) Ninety‐four polymorphic AFLP markers distinguished two population clusters defined largely by samples on and off the Colorado Plateau; (b) substructure of samples on the Colorado Plateau was indicated by genetic divergence between boundary and interior regions, and was supported by cytotype distribution and cpDNA analysis; and (c) six AFLP markers were identified as “outliers,” consistent with being under selection. These loci were significantly correlated to mean annual temperature, mean annual precipitation, precipitation of driest quarter, and precipitation of wettest quarter in natural populations, but not in cultivated samples. Marker × environment relationships were found to be largely influenced by cytotype and cultivar development. Our results demonstrate that blue grama is genetically variable, and exhibits genetic structure, which is shaped, in part, by environmental variability across the Colorado Plateau. Information from our study can be used to guide the selection of seed source populations for commercial development and long‐term conservation management of *B. gracilis*, which could include genetic assessments of diversity and the adaptive potential of both natural and cultivated populations for wildland restoration.

## 1 INTRODUCTION

It is becoming increasingly important to understand how environmental factors shape genetic variation in plants, especially in the southwestern United States where climate change is expected to have major impacts (Archer & Predick, 2008; Gremer, Bradford, Munson, & Duniway, 2015). Such information is critical for species that are commonly used for restoration, such as grasses, which are often dominant components of ecosystems on the Colorado Plateau. Given their broad distribution, local adaptation is likely to play a key role in how grasses will respond to climate change (Alberto et al., 2013; Davis & Shaw, 2001; Jump & Penuelas, 2005) and may be predictive of the success of cultivated seeds for restoration purposes (Hufford & Mazer, 2003; Linhart, 1995; Mijangos, Pacioni, Spencer, & Craig, 2015).

Due to anthropogenic activity, drastic and widespread changes to ecosystems have and will continue to occur (International Panel on Climate Change, 2014; Parmesan & Yohe, 2003; Vitousek, Mooney, Lubchenco, & Melillo, 1997). Many of these changes are also accompanied by uncharacteristic disturbance regimes (Dale et al., 2001; Flannigan, Stocks, & Wotton, 2000). Because local adaptation is so widespread among plant species, a changing climate is likely to influence all species, even those whose distributions span large environmental gradients (Davis & Shaw, 2001; Reusch & Wood, 2007). It is unclear, however, whether species distributed across these gradients will persist through natural dispersal alone (Jump & Penuelas, 2005), perhaps warranting assisted migration in the near future. Information on genetic‐based adaptation in widespread grasses is particularly critical given the increased emphasis on restoration of wildlands, which are currently under accelerated change due to anthropogenic forces such as climate change, fire, and habitat disturbance. In the southwestern United States, for example, increased fire frequency in grasslands drives increased demand for seed. The average acres burned per year by wildland fires has nearly tripled since the 1980s, with an average of 7.2 million acres burned annually in the United States between 2000 and 2007, with a particularly alarming increase in the western United States (National Interagency Fire Center, 2015).

Although its efficacy is debated, reseeding is extensively used to reduce soil erosion, suppress non‐native plants, and restore desirable plant communities following high‐intensity wildfires (Beyers, 2004; Burton & Burton, 2002). Cost associated with reseeding burned areas has also substantially increased in recent years: The Forest Service Burned Area Emergency Response (BAER) seeding expenditure alone has reached an average of $3.3 million per year, which is nearly triple the average annual expense of the previous 30 years (Peppin, Fule, Sieg, Beyers, & Hunter, 2010). Such high costs could be more defensible by thoroughly understanding the effectiveness of restoration activities. One way to address such concerns is to investigate the degree to which source populations used for restoration purposes, including both natural and cultivated sources, are locally adapted to their environment. A first step toward investigating adaptive potential in these populations can be achieved by assessing genome‐wide variability and determining the extent to which this variability is influenced by environmental variation.

A key issue for conservation management concerns the degree to which cultivated varieties maintain similar levels of genetic variability and structure as wild source populations. Although seed sources that are locally adapted have consistently been recognized for their increased success for restoration purposes (Langlet, 1971; Leimu & Fischer, 2008; Lesica & Allendorf, 1999), cultivated varieties are presumably less genetically varied than their wild counterparts. Because of this, there is growing concern that cultivated varieties widely used in large‐scale restoration can be futile or even detrimental to meeting land management objectives. Many cultivated varieties, for example, may have adapted to constant human care with frequent selection for large biomass and high seed yield. These potential cultivation pressures may lead to the loss of traits that allow for survival in a variable wildland climate (Coyne & Lande, 1985). Cultivar varieties may outcompete or hybridize with locally adapted plants, potentially causing native populations to lose important genetic traits that enable them to respond to natural fluctuations in their local environments (Schröder & Prasse, 2013a, 2013b). Thus, understanding the extent of genetic variability in widespread species and cultivated varieties and how it is structured across broad environmental gradients can provide important clues for conservation management.

Here, we assess genetic variability, structure, and the potential for local adaptation in the native grass species, *Bouteloua gracilis,* through the analysis of allelic variation within and among populations across a broad environmental gradient. Allelic variance was coupled with spatial and environmental variables (Manel et al., 2010; Schoville et al., 2012) to determine relationships between potentially adaptive “outlier” loci and source environment for individual populations (Beaumont & Balding, 2004; Riebler, Held, & Stephan, 2008).

To better understand how environment, cytotype, and cultivation shape genetic variation in *B. gracilis*, we addressed the following questions:

1. How genetically diverse is *B. gracilis* in the Colorado Plateau region and how is genetic variation structured across populations? Are phylogenetic relationships of these populations similarly structured?
2. Does population genetic structure in *B. gracilis* correlate with key climatic variables (e.g., temperature and precipitation) and how do these relate to adaptation to specific environments?
3. Does genetic structure and variability covary with cytotype? If so, is it linked to differentiation of functional traits as suggested for other species (Chao et al., 2014; Khazaei, Monneveux, Hongbo, & Mohammady, 2010; Manzaneda et al., 2011; Yang, Huang, Quin, Zhao, & Zhou, 2013), Madlung (2013)?
4. Are cultivated varieties of *B. gracilis* genetically distinct from and less genetically diverse than natural populations? We consider this to be a critical question because locally adapted seed has consistently been recognized for its increased success for restoration (Langlet, 1971; Leimu & Fischer, 2008; Lesica & Allendorf, 1999), while many cultivars have demonstrated susceptibility to agricultural pressures that lead to loss of traits that allow for survival in a variable climate (Schröder & Prasse, 2013a, 2013b).

## 2 METHODS

### 2.1 Study site

The Colorado Plateau is approximately 362,600 square kilometers in the Four Corners region of Arizona, Utah, Colorado, and New Mexico. It is characterized by diverse climates that range from Sonoran Desert to Alpine, with elevations ranging from 914 to 4,267 m. The region is dominated by semiarid conditions and includes the watersheds of the Colorado River and its tributaries including the Green, San Juan, and Little Colorado Rivers (Foos, 1999).

Annual precipitation is broadly varied on the Colorado Plateau. Though the average annual precipitation across the region is 254 mm (Foos, 1999), the minimum average rainfall is 136 mm, while areas above 2,440 m in elevation receive over 508 mm and mountaintops over 3,353 m can receive nearly 1 m (U.S. Bureau of Land Management, 2014). Seasonal monsoon activity is also widespread across the region, with a significant north–south and east–west trend in precipitation seasonality (PS), with the monsoon being stronger in the southern and eastern portions of the Plateau (Adams & Comrie, 1997). Temperatures are also highly variable, with southern and lower elevation temperatures ranging from −7°C in the winter to 35°C in the summer, and mid and upper elevation temperatures ranging from −20°C in the winter to 15–25°C in the summer. The region also undergoes multidecadal drought cycles that are associated with the Pacific Decadal Oscillation (USBLM, 2014).

### 2.2 Study species

*Bouteloua gracilis* is a highly valued species for conservation and restoration (Herbel, Steger, & Gould, 1974) because of its broad range, adaptability, ease of establishment, and year‐round forage value for livestock and wildlife (Morris, Booth, Payne, & Stitt, 1950). It is a densely tufted, C4 perennial grass that is widespread from Alberta and Manitoba, Canada, south through the Rocky Mountains and Great Plains, Midwest United States, and northwestern México (Cronquist, Holmgren, Holmgren, Reveal, & Holmgren, 1977). Blue grama inhabits a wide array of habitat types including sagebrush, salt‐desert shrub, oak woodland, ponderosa pine, pinyon‐juniper, prairies, and montane (Anderson, 2003) and is regularly a dominant species in the ponderosa pine and pinyon‐juniper ecosystems of the Colorado Plateau.

*Bouteloua gracilis* is predominantly outcrossing, but clonal propagation also occurs following establishment by seed (Miller, 1967a; Riegel, 1941). Seeds are dispersed short distances by wind (Coffin & Lauenroth, 1989) and insects (Wicklow, Rabinder, & Lloyd, 1984), and longer distances by adhesion to animals (Sorensen, 1986). Fragmented populations typically have few prospects for long‐distance seed dispersal and may exhibit restricted gene flow (Anderson, 2003), though gene flow could be expected to remain high even in patchy environments due to long‐distance wind dispersal of pollen (Llorens et al., 2016). *Bouteloua gracilis* is also evidently an autopolyploid across the study region, with diploid, tetraploid, and mixed cytotype sites distributed across the Colorado Plateau (Butterfield & Wood, 2015).

### 2.3 Collections

**Figure 1:**
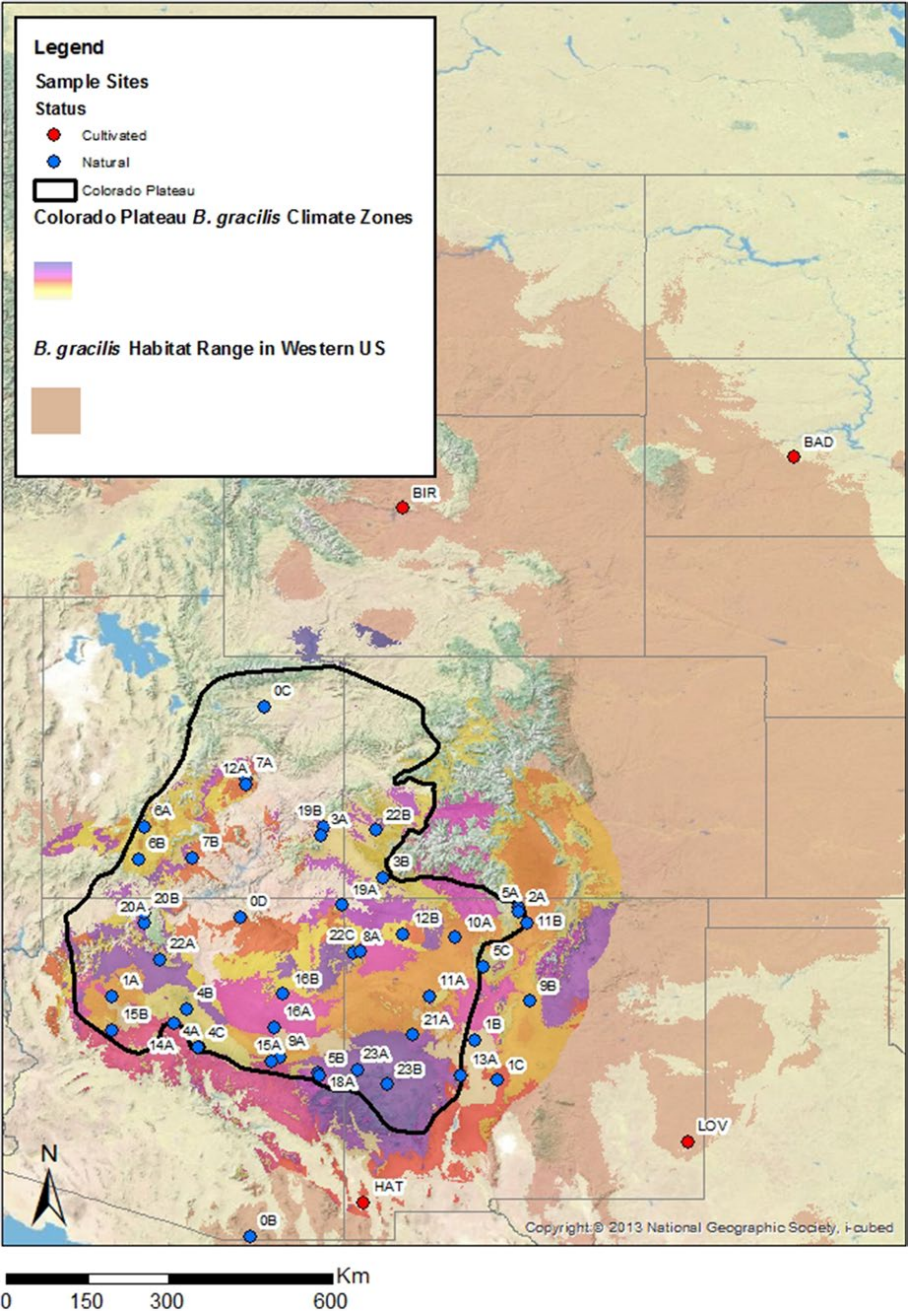
Map of sampling site distribution and climate zones. Natural sampling sites (blue), cultivar source locations (red), and *Bouteloua gracilis*‐specific climate zones across the Colorado Plateau and adjacent regions as identified by the climate modeling method defined by Doherty et al. (2017)

Forty‐four collection sites (Table 1, Figure 1) were selected as representative of the species distribution both geographically and climatically. To establish a representative distribution to capture climate variability, the species’ geographic range was coupled with data obtained from WorldClim (Hijmans, Cameron, & Parra, 2005), including annual mean temperature, mean diurnal range, isothermality, temperature seasonality, maximum temperature of the warmest month, minimum temperature of the coldest month, temperature annual range, mean temperature of the wettest/driest and warmest/coldest quarter, annual precipitation, precipitation of the wettest/driest month, PS, and precipitation of the wettest/driest and warmest/coldest quarter. This information was used to differentiate 25 climate zones within the range of *B. gracilis* across the Colorado Plateau and adjacent regions (using spatial‐climatic stratification methods described in Doherty, Butterfield, & Wood, 2017). Twenty‐three of 25 zones were sampled. Approximately two sample sites within each zone were selected, and the resulting sample site distribution was geographically varied. Sample sites are identified with the zone as the prefix (0, 1, 2, etc.), followed by an alphabetic identifier (A, B, C, etc.) to differentiate multiple sample sites within a single climate zone. Sites that begin with “0” do not indicate occupation of similar climate zones, but rather a region that fell outside of delineated climate‐model zones.

**Table 1:** Sampling site location information

Population ID	City/Landmark	State	CO Plateau locality	Latitude	Longitude	Ploidy		
						2×	4×	6×
0B	Patagonia	AZ	South of Rim	31.38	−110.58		0.9	0.1
0C	Duchesne	UT	North Rim	40.17	−110.36			
0D	Black Mesa	AZ	Interior	36.67	−110.75		0.9	0.1
1A	Seligman	AZ	West Rim	35.36	−112.89		1.0	0.0
1B	Belen	NM	South East of Rim	34.64	−106.86			
1C	White Sands Desert	NM	South East of Rim	33.97	−106.49	1.0		
1D	White Bluffs	NM	South East of Rim	35.28	−105.95			
2A	Tusas Mountains	NM	East Rim	36.75	−106.14	1.0		
3A	Hats Draw	UT	Interior	38.03	−109.42	0.1	0.9	
3B	Cortez	CO	East of Rim	37.33	−108.38			
4A	Mogollon Rim	AZ	South Rim	34.52	−111.44		1.0	
4B	Flagstaff	AZ	South Rim	35.14	−111.64	0.2	0.8	
4C	Mogollon Rim	AZ	South Rim	34.50	−111.45		1.0	
5A	Broke Off Mountain	NM	East Rim	36.82	−106.15	1.0		
5B	Antelope Vista	AZ	South Rim	34.08	−109.46			
5C	Jemez Mountains	NM	East Rim	35.86	−106.74	1.0		
6A	Circleville Mountain	UT	Northwest Rim	38.17	−112.35	1.0		
6B	Hatch Valley	UT	West Rim	37.63	−112.44			
7A	San Rafael Swell	UT	Interior	38.98	−110.67		1.0	
7B	Grand Staircase	UT	Interior	37.66	−111.54			
8A	Chuska Mountains	AZ	Interior	36.09	−108.87			
9A	Taylor	AZ	South Rim	34.35	−110.11			
10A	Lindrith	NM	Interior	36.33	−107.19			
11A	San Mateo	AZ	South East Rim	35.36	−107.62		1.0	
11B	Kiowa Mountain	NM	East Rim	36.58	−106.01		0.9	0.1
12A	Zuni Plateau	UT	Interior	38.87	−110.66		1.0	
12B	Petrified Forest	AZ	Interior	36.38	−108.06			
13A	South Baldy	NM	South East of Rim	34.05	−107.11			
14A	Red Rocks	AZ	South of Rim	34.90	−111.86	0.1	0.9	
15A	Mogollon Rim	AZ	South Rim	34.28	−110.24		1.0	
15B	Pine Creek	AZ	South Rim	34.80	−112.89			
16A	Holbrook	AZ	Interior	34.84	−110.19	0.5	0.5	
16B	Hopi Volcanic Field	AZ	Interior	35.39	−110.05		1.0	
18A	Greer	AZ	South Rim	34.03	−109.46			
19A	Twin Falls	AZ	Interior	36.87	−109.08			
19B	La Sals Flatlands	UT	Interior	38.17	−109.38			
20A	Lookout Canyon	AZ	West Rim	36.59	−112.35	1.0		
20B	Snake Gulch	AZ	West Rim	36.73	−112.36		1.0	
21A	Cebollita Valley	AZ	South East Rim	34.71	−107.90	0.1	0.9	
22A	Tusayan	AZ	West Rim	35.96	−112.10		1.0	
22B	Naturita Draw	CO	East Rim	38.11	−108.51			
22C	Sheep Springs	AZ	Interior	36.11	−108.77			
23A	Cerro La Muna	AZ	South Rim	34.14	−108.81		1.0	
23B	Horse Mountain Basin	NM	South Rim	33.90	−108.33			
ALM	Alma	NM/KS/TX	Cultivar‐Composite	NA	NA			
BAD	Bad River	SD	Cultivar	44.30	−101.57			
BIR	Bird's Eye	WY	Cultivar	43.46	−108.07			
HAT	Hatchita	NM	Cultivar	31.94	−108.71			
LOV	Lovington	NM	Cultivar	32.95	−103.34			

Note

Population ID is defined by *Bouteloua gracilis*‐specific Colorado Plateau climate zone number (Doherty et al., 2017) and subsequent assignment of A, B, C, or D to differentiate between separate sampling occurrences within a single climate zone. Population IDs beginning with “0” are an exception and do not indicate a shared climate zone but rather indicate a site that fell outside of the expected *B. gracilis* range based on climate data. Latitude and longitude are formatted in decimal degrees and are projected using WGS 84. Ploidy is indicated as the proportion of sampled individuals within each collection site that is diploid (2×), tetraploid (4×), or hexaploid (6×). Ploidy fields are grayed out if no information is available for any given ploidy race at a site.

In addition to the 44 sampling locations of native populations, five *B. gracilis* cultivars developed for restoration were also selected for analysis. Bad River and Bird's Eye are recommended for use across the Great Plains, while Lovington, Hatchita, and Alma are heavily used for restoration on the Colorado Plateau. Lovington was released in 1962 and was sourced in 1944 from southeastern New Mexico. It was selected for increased forage production and is recommended for planting only in regions receiving 12 inches or more of annual rainfall (United States Department of Agriculture, 1964). Hatchita was released in 1982, originally sourced from southwestern New Mexico, and selected for increased seedling vigor and drought tolerance (USDA, 1982). Alma was released in 1992, traces to Lovington, Hatchita, and an experimental composite (Texas, Kansas), and was selected for seedling vigor and wide adaptability (USDA, 1992).

Samples for this study were derived from both natural sites and collections that were obtained from natural sites, but maintained locally either in a common garden or greenhouse for approximately 1 year. Thus, all samples were originally sourced from the wild, with the exception of the cultivars, which were obtained as germplasm (seed) and subsequently germinated to obtain leaf tissue. One hundred and thirteen samples were obtained from 17 natural sites. One hundred and ten samples were obtained from a common garden representing 20 natural sites. Twelve samples from two natural sites were obtained from living specimens growing at the Northern Arizona University greenhouse in October of 2015. Forty‐four samples from six natural sites were grown from seed in December of 2015. Eighty‐one samples from five cultivated varieties were also grown from seed (~16 samples/cultivar). All materials were harvested while still green, desiccated upon collection in science‐grade silica and stored at room temperature.

As indicated above, some samples were obtained from specimens grown from seed collected by the Seeds of Success (SOS) program following their standardized protocol and distributed by the SOS program directly or the Germplasm Resources Information Network. All cultivar seeds were obtained from the Granite Seed Company distribution center in Lehi, UT. Granite Seed is a heavily utilized distributor of native seed for large‐scale restoration purposes. While the specific agricultural production site of the seeds remains undisclosed by Granite Seed, the varieties and state of origin include Hatchita (CO), Lovington (CO), Alma (CO), Bird's Eye (WY), and Bad River (MN), with the first three being the primary varieties utilized on the Colorado Plateau.

### 2.4 Cytotype distribution and ploidy measurements

Cytotypic distribution, including diploids, tetraploids, and a few hexaploid sites, has been identified across the Colorado Plateau for *B. gracilis *(Table 1). Of the 44 sites included in the amplified fragment length polymorphism (AFLP) analysis of this study, 26 have cytotype identified. Ploidy was determined using flow cytometry of fresh leaf material provided by the U.S. Forest Service's Provo Shrub Sciences Lab in Provo, UT (specified in Butterfield & Wood, 2015).

### 2.5 DNA extraction

DNA was extracted from approximately seven individuals from each of the 44 natural sites and 15 specimens for each of the five cultivated varieties (for a total of 360 individuals) using the MagJET reagents and protocol as outlined in “The User Guide: MagJET Plant Genomic DNA Kit” (Thermo Fisher Scientific). Five no‐template controls (NTCs) and 25 replicated samples were included. Replicates consisted of same‐plate and different‐plate individuals to identify any possible plate effects on subsequent analysis.

### 2.6 AFLP analysis

All extracted samples were analyzed using AFLP genotyping using a protocol slightly modified from Gray et al. (2014). Restriction and ligation reactions were done simultaneously in 30 μl reaction volumes and were composed of 150 ng of extracted DNA (20 μl total volume with suspension in Tris‐Cl pH 8.0, 7.5 ng of DNA/μl), ddH_2_O (4.8 μl), BSA (0.2 μl of 20 mg/ml), 500 units of T4 ligase (0.25 μl of 2,000 units/μl concentration), T4 buffer (2 μL of 10× concentration), EcoRI adapter (1 μl of 5 pM/μl concentration), MseI adapter (1 μl of 50 pM/μl concentration), and 10 units each of EcoRI (0.25 μl of 40 units/μl concentration) and MseI (0.5 μl of 20 units/μl concentration). Reactions remained at room temperature overnight for approximately 18 hr and were diluted 1:10 in Tris‐Cl, pH 8.0.

Preselective and selective polymerase chain reactions (PCRs) were run in 10 μl reaction volumes and were composed of 2× Phusion Green HSII High‐Fidelity PCR Master Mix by Thermo Fisher (5 μl), MgCl_2_ (0.3 μl of 50 mM), ddH_2_O (3.45 μl), a primer combination (0.25 μl of 20 μM concentration), and DNA template (1 μl). Preselection product was diluted 1:20 in Tris‐Cl, pH 8.0 prior to selective PCR. Selective PCR product was diluted 1:10 in ddH_2_O and run on a 3730 DNA Analyzer (Thermo Fisher).

Thermal cycler conditions were as follows for the preselective PCRs:20°C for 5 s; ramp from 20 to 70°C (0.2°C/s); 70°C for 2 min; 94°C for 1 min; then 30 cycles of 94°C for 30 s, 56°C for 1 min; 72°C for 1 min; followed by 72°C for 10 min; 15°C for 5 min; and a hold at 4°C.

Thermal cycler conditions for the selective PCRs were as follows: 95°C for 2 min; 13 cycles of 65°C for 30 s (−0.7°C/cycle), 72°C for 90 s, and 94°C for 30 s; then 23 cycles of 56°C for 30 s, 72°C for 90 s, and 94°C for 30 s; followed by 72°C for 10 min, 15°C for 5 min, and a hold at 4°C.

A total of 12 selective AFLP primer combinations were screened, with a panel of six geographically representative samples, including replicates of all six samples. Of these, three primer combinations were chosen based on replicability and consistency: M49/E35, M50/E35, and M62/E35 (Table 2). These selective primer combinations relied on template produced from the PCR preselection amplification using primer combination M02/E01.

**Table 2:** Primers used for amplified fragment length polymorphism (AFLP) analysis

AFLP primer sequences			
No.	Primer	Code	Sequence 5' to 3'
1	AFLP: PreSelective (+1)	E01	GACTGCGTACCAATTCA
2	AFLP: PreSelective (+1)	M02	GATGAGTCCTGAGTAAC
3	AFLP: Selective (+3)	E35	GACTGCGTACCAATTCACA
4	AFLP: Selective (+3)	M49	GATGAGTCCTGAGTAACAG
5	AFLP: Selective (+3)	M50	GATGAGTCCTGAGTAACAT
6	AFLP: Selective (+3)	M62	GATGAGTCCTGAGTAACTT

Note

Standard oligonucleotide primers were used to assess nuclear DNA variability through AFLP analysis. The preselective primer combination M02‐E01 was used for all subsequent selective PCR amplifications. Final selective primer combinations used include E35‐M49, E35‐M50, and E35‐M62.

Electropherograms were analyzed using GeneMarker (SoftGenetics) software. Panels were manually created, and loci were automatically scored and manually confirmed as present (1) or absent (0) for each sample. GeneMarker parameters included smoothing, inclusion of fragment lengths of 50–500 base pairs, a peak height threshold of 50, local and global detection percentages set to off, and the stutter peak filter set to off. Percent error rates were calculated by comparing reproducibility and consistency among replicates as a whole and at each locus.

### 2.7 Chloroplast DNA sequencing

To provide a framework for examining patterns detected by the AFLP analysis and to investigate colonization history, chloroplast DNA (cpDNA) sequences were acquired from select samples using the Sanger‐sequencing method. Seventeen natural sites and all five cultivars, each represented by a single individual, were used for the cpDNA analysis. Eight primer combinations (Shaw et al., 2005) were screened across four geographically diverse samples. Of those, four successfully amplified the expected product (including psbA‐trnH, 3′trnG–5′trnG2G, rps16, and rpoB‐trnC), with the former two producing polymorphisms that were applied across the broader subset of samples.

DNA templates were suspended in Tris‐Cl pH 8.0 and standardized to 5 ng/μl. Polymerase chain reactions (PCRs) were run in 10 μl reaction volumes and were composed of 2× Phusion Green HSII High‐Fidelity PCR Master Mix by Thermo Fisher (5 μl), MgCl_2_ (0.3 μl at 50 mM), ddH_2_O (3.6 μl), a primer combination (0.1 μl at 200 nM concentration), and template (1 μl). Thermal cycler conditions were as follows: 95°C for 1 min; then 35 cycles of 95°C for 1 min, 54°C for 30 s, and 65°C for 2 min; followed by 72°C for 5 min and a hold at 4°C. PCR product was purified using a 1:1 18% PEG magnetic bead clean‐up and rinsed twice with 70% ethanol.

Cycle‐sequencing reactions were run in 10 μl reaction volumes and were composed of Applied Biosystems Big Dye Terminator (0.5 μl), MgCl_2_ (0.3 μl at 50 mM), ddH_2_O (to volume), a single primer (2 μl at 15 mM concentration), and template (3 ng/100 base pairs expected; approximately 3.5 μl). Both forward and reverse primers were cycled independently to create consensus sequences. Thermal cycler conditions were as follows: 95°C for 2 min; then 50 cycles of 95°C for 10 s, 50°C for 10 s, and 60°C for 2 min; followed by a 10°C hold. Cycle‐sequencing products were purified using a 1:3 25% PEG magnetic bead clean‐up, rinsed twice with 70% ethanol, and sequenced on a 3730 Genetic Analyzer. Electropherograms were imported into Staden (Bonfield, Smith, & Staden, 1995) to generate and assess quality of consensus sequences and subsequently aligned in BioEdit (Hall, 2004) using the accessory application ClustalW Multiple Alignment on default settings.

### 2.8 Statistical analysis

Genetic distance calculations, allelic frequency measures, and analysis of molecular variance (AMOVA) were performed in GenAlEx (Peakall & Smouse, 2006). Structure (Pritchard, Stephens, & Donnelly, 2000; ver. 2.3) was used to identify statistically significant population structure through the identification of population clusters or *K* (Porras‐Hurtado et al., 2013). The analysis was run using the admixture (with location priori) and the independent allele model options with a burn‐in of 10,000, a run length of 30,000, and five iterations, which resulted in stabilized parameters and no change in log‐likelihood values with increased replication. Structure Harvester (Earl & vonHodt, 2012) was used to estimate the most probable value for *K* based on the Evanno method (Evanno, Regnaut, & Goudet, 2005). CLUMPP (Jakobsson & Rosenberg, 2007) was used to correct for label switching and multimodality of the data. Lastly, the CLUMPP output was graphically illustrated using Excel. The analysis was performed including and excluding outlier loci (see BayeScan below), with negligible differences detected.

The program BayeScan 2.01 (Foll & Gaggiotti, 2008) was used to detect outlier loci. BayeScan can directly estimate the probability that each locus is under selection by identifying population and marker‐specific components of *F*_ST_ values using a logistic regression. A burn‐in of 50,000 was used with chains of 1,000,000 iterations. The posterior probability was set to 0.76 and above, and the false discovery rate was set to 5%. The remaining parameters were defaulted including 20 pilot runs of 5,000 iterations in length, prior odds for the neutral model of 10, and a uniform distribution of *G*_ST_ (analog of *F*_ST_) between 0 and 1 (Henry & Russello, 2013). BayeScan identified six putative outlier loci, potentially under selection. These are identified by their individual fragment sizes in base pairs: 193, 286, 378, 407, 436, and 471 bp.

All 19 bioclimatic variable values were obtained from WorldClim (Hijmans et al., 2005). Redundancy of variables was identified if any had a correlation value of 0.8 or greater. The variable considered most biologically relevant was kept and the other discarded. Additionally, if the correlation of a particular variable was closely reflected in another variable across all markers, again only the most biologically relevant was kept and the other discarded. Of the 19 environmental variables procured through WorldClim, six were identified as nonredundant and considered in more detail: mean annual temperature (MAT), temperature seasonality, mean annual precipitation (MAP), PS, precipitation of driest quarter (PDQ), and precipitation of the coldest quarter. All six outlier loci were correlated with one or more of these environmental variables.

To assess environmental and outlier loci frequency relationships, general linear models and correlation analyses (Table 3) were conducted in R and verified using the Analysis Toolpak in Excel. Because unequal sample sizes were an issue in population and cytotype comparison, Fisher‐*Z* statistics (Meng, Rosenthal, & Rubin, 1992) were used to test the comparison of correlations derived from independent samples. The influence of geographic distance on outlier loci was examined using Mantel and partial‐Mantel tests (Legendre & Legendre, 1998; Mantel, 1967).

**Table 3:** Correlation coefficients of outlier loci, categorical groupings, and environmental variables for natural sampling sites

Correlation coefficients of natural sampling sites							
AFLP markers		Environmental variables					
Primer Combo	Locus (fragment length)	Mean annual temp	Temp seasonality	Annual precip	Precip seasonality	Precip of driest quarter	Precip of coldest quarter
M49‐E35	378***	0.31***	0.20**	0.24**	0.18*	0.36***	0.27**
M49‐E35	407***	0.18*	0.13*	0.20**	0.15*	0.14*	0.27**
M49‐E35	471***	0.07*	0.26	0.30**	0.00*	0.23	0.28**
M50‐E35	436***	0.21**	0.05*	0.11*	0.16*	0.11*	0.18*
M60‐E35	193***	0.30**	0.03*	0.11*	0.14*	0.18*	0.11*
M60‐E35	286***	0.26**	0.07*	0.21**	0.23**	0.28**	0.27**
Average correlation		0.21**	0.14*	0.20**	0.13*	0.22**	0.24**

Categorical groups	Mean annual temp	Temp seasonality	Annual precip	Precip seasonality	Precip of driest quarter	Precip of coldest quarter
AFLP clades	0.42***	0.13*	0.36***	0.46***	0.41***	0.52***
Cytotype	0.42***	0.01*	0.27**	0.38***	0.62***	0.34***

Note

Correlation values of outlier amplified fragment length polymorphism (AFLP) loci to environmental values (top) as well as correlation values of categorical groups (as defined by AFLP data and by cytotype) and environmental variables (bottom). *** Indicates correlation coefficients > 0.3; ** Indicates correlation coefficients between 0.2 and 0.3; * Indicates correlation coefficients < 0.2.

Molecular evolutionary genetic analysis, or MEGA version 5.1 (Tamura et al., 2011), was used for generating phylogenetic trees, including distance‐based neighbor‐joining and UPGMA trees to examine population genetic clustering as indicated by AFLP loci. Phylogenetic trees were generated for cpDNA sequences using maximum‐likelihood, maximum parsimony, and neighbor‐joining methods. For all three trees generated from the cpDNA data, 1,119 positions were identified including 24 variable sites. Positions containing gaps or missing data were omitted. Bootstrap values were calculated with 1,000 iterations (Felsenstein, 1985). For the cpDNA neighbor‐joining tree, evolutionary distances were computed using the maximum composite likelihood (MCL) method (Tamura, Nei, & Kumar, 2004) and are in the units of the number of base substitutions per site. The maximum parsimony tree was generated with the subtree‐pruning‐regrafting algorithm (Takahashi & Nei, 2000) with search level 1, in which the initial trees were obtained by the random addition of sequences (10 replicates). The maximum‐likelihood method, using cpDNA data, was based on the Tamura‐Nei model (Tamura & Nei, 1993). Initial trees for the heuristic search were obtained automatically by applying Neighbor‐Joining and BioNJ algorithms to a matrix of pairwise distances estimated using the MCL approach and then selecting the topology with the superior log‐likelihood value. Because all three methods produced similar results, only the neighbor‐joining tree is presented.

## 3 RESULTS

### 3.1 Genetic variability and structure

Collectively, the three AFLP primer combinations yielded 94 polymorphic loci. Each primer combination yielded an average of 32 polymorphic loci, an average error rate of 4.2% for individual loci, and an average error rate of 2.9% across replicates. Although the number of polymorphic loci is relatively low for the AFLP method, our results are consistent with other *B. gracilis* studies, including one resulting in 167 polymorphic loci and an average of 28 bands per primer combination (Fu, Ferdinandez, Phan, Coulman, & Richards, 2003) and one RAPD study that resulted in 69 polymorphic loci with an average of only six bands per primer combination (Phan, Fu, & Smith, 2004). As was also found in these studies, our results did not identify any locus/allele that was unique to a single population or seed source. For populations derived from natural sites, average percent polymorphism (%P) was 43.96 and ranged from 10.64 to 77.66; for cultivated varieties, %P averaged 69.57 and ranged from 64.89 to 74.47. For natural populations, average expected heterozygosity (*H*_e_) was 0.174 and ranged from 0.003 to 0.302; for cultivated varieties, *H*_e_ averaged 0.258 and ranged from 0.232 to 0.286. Half of the populations’ average *H*_e_ fell close to or near (±0.03) the average *H*_e_. Sites with above‐average *H*_e_ were found within three of the four natural sampling sites for the off‐Colorado Plateau region. Four additional above‐average *H*_e _sites were identified across the central portion of the Plateau. Eleven sample sites had below‐average *H*_e,_ and all occurred within the Colorado Plateau region. Six of these sites dominated the northern region of the Plateau, with particularly high occurrence near the boundary of the Plateau, while the remainder occupied the more central portions of the Plateau's boundary.

**Figure 2:**
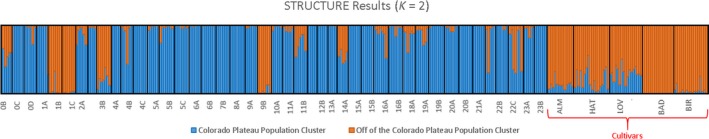
Population clusters (*K* = 2) as identified by Structure. All cultivar varieties strongly cluster with natural populations 1B, 1C, 3B, and 9B. These populations, as well as the cultivar source populations, are all on the far eastern edge of or off the Colorado Plateau and are referred to as the “Off the Colorado Plateau Population Cluster” (orange) in the text. All other natural populations are predominantly on the Colorado Plateau (with the exception of 0B, 6A, and 6B) and are therefore referred to as the “Colorado Plateau Population Cluster” (blue)

Two genetically distinct populations were identified using the program Structure: one largely on the Colorado Plateau and the other off the Colorado Plateau (Figure 2). All five cultivated varieties were included in the off‐Plateau group, which aligns with the fact that all the cultivars were originally sourced from natural populations located off the Colorado Plateau.

When all 44 natural populations were analyzed together, the AMOVA showed that 17% of genetic variation was attributed to differentiation between the two regions: on and off the Colorado Plateau (Table 4). Approximately 13% was due to differences between populations within each region, and 70% was attributed to differences among individuals within populations. The off‐Plateau group had greater population structure than the Plateau group, with PhiPT values of 0.22 and 0.15, respectively. Only 2% of the genetic variance within the off‐Plateau group could be attributed to differences between the natural populations and the cultivars, indicating that the cultivar varieties reflect similar levels of genetic variation present within the species’ natural populations.

**Table 4:** AMOVA of AFLP data

Regions	Within populations	Among populations	Among regions
Colorado Plateau, off‐Plateau sites (including cultivars)	0.70	0.13	0.17
Off‐Plateau sites, cultivars	0.81	0.17	0.02
Colorado Plateau sites	0.85	0.15	
Northern, southern cultivars	0.86	0.08	0.06
Diploids, tetraploids	0.74	0.17	0.09

Note

All AMOVA analyses were based on Nei's genetic distance calculated from AFLP data. Sampling sites were standardized at *n* = 5 individuals, though AMOVA runs with unequal and/or larger sample sizes did not result in significantly different results.

AFLP: amplified fragment length polymorphism; AMOVA: analysis of molecular variance.

When assessing cultivars independently, 11% of their genetic variation was due to differences among individual varieties, with 6% of genetic variation due to differences between the two Northern Plains varieties (Bad River, Bird's Eye) and the three southwestern varieties (Alma, Lovington, Hatchita). Although the AMOVA analysis showed nominal differentiation between the off‐Plateau group and cultivars (2%), the frequencies of three of the six outlier loci (193, 378, and 436 bp) significantly (*p* < 0.05) differed between these two groups, ranging from 0.11 and 0.19, with an average of 0.15. The differentiation between the Colorado Plateau population from both the cultivars and natural off‐Plateau population was even more extreme, with high significance (*p* < 0.006) in regard to outlier loci frequency across all six markers, with a change in locus frequency ranging from 0.27 to 0.55, with an average of 0.4. Furthermore, three of the six loci were present in the cultivar individuals at a frequency of 100% (i.e., fixed), while none of the natural populations demonstrated genetic fixation of any identified outlier loci for any of the environmental variables examined.

### 3.2 Phylogenetic relationships based on AFLPs

**Figure 3:**
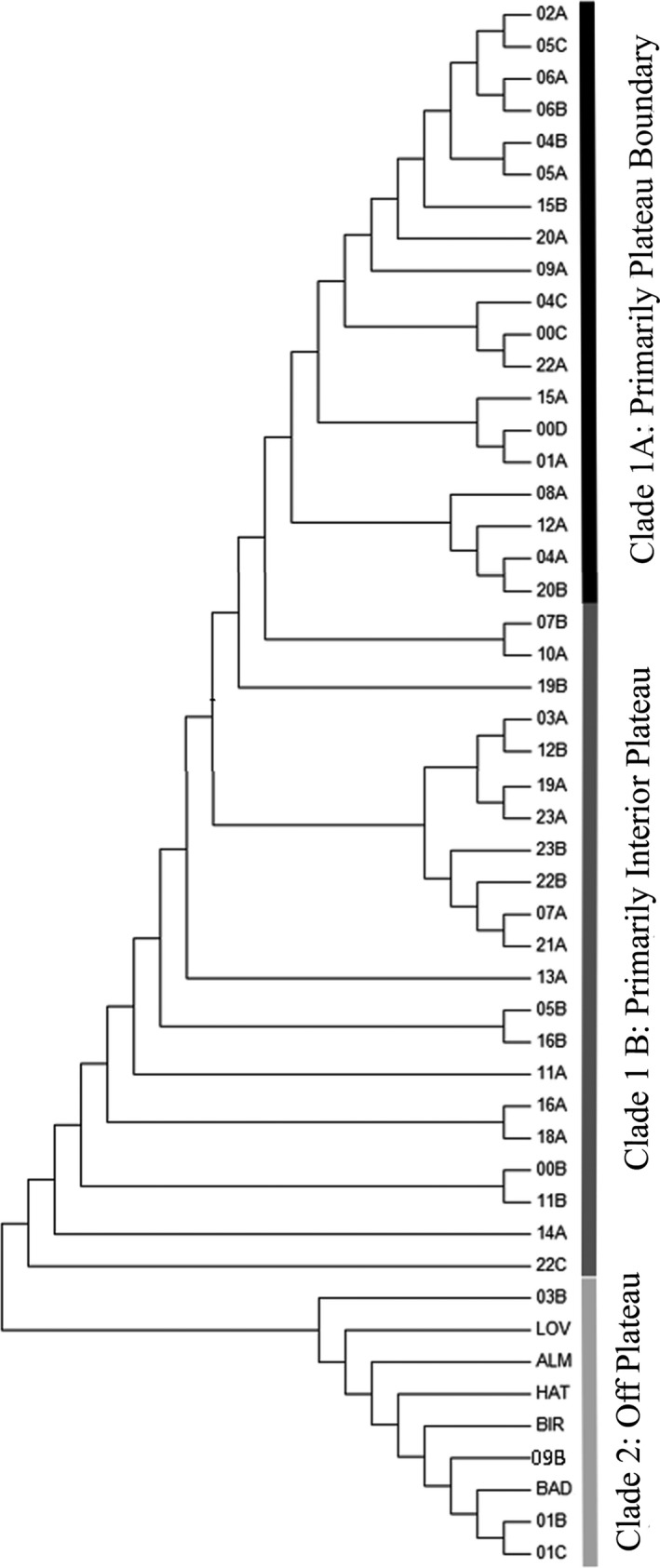
Neighbor‐joining phylogenetic tree as indicated by amplified fragment length polymorphism (AFLP) data. The evolutionary distances are based on the computed genetic distance of sites (GenAlEx). Like MEGA, the program Structure also identified Clade 1 as distinct from Clade 2

A neighbor‐joining analysis of the AFLP data revealed two major clades (Figure 3), consistent with the two groups identified by Structure (on and off the Colorado Plateau). Assessment of relationships identified on the Colorado Plateau indicated a broad divergence of boundary (Clade 1A) and interior sites (Clade 1B). The eight most distantly related sampling sites from the off‐Plateau population (Clade 1A) share several features: They are all on the eastern (five sites) or western (three sites) flanking boundary; and they are likely all diploid (five of eight sites were identified as diploid, while three sites remain uncharacterized). The remaining 11 sampling sites of Clade 1A are notably tetraploid and are also all largely located on or near the western boundary (though there is an evident lack of sampling along the eastern boundary). The exceptions to this trend are sampling sites 0D and 8A, which occur centrally on the Colorado Plateau. These sites are both on western sides of the dominant elevational features of the central Colorado Plateau (Black Mesa and Chuska Mountains, respectively) that may reflect similar abiotic factors of those sites occupying the Colorado Plateau boundary region. The remaining sampling sites of the Colorado Plateau population (Clade 1B) reside predominantly in the interior of the Plateau or on the southeastern boundary adjacent to the nearest off‐Plateau sampling sites. Of the 21 sample sites of Clade 1B, 10 have cytotype identified: four are tetraploid and six are mixed ploidy sites. The off‐Plateau population (Clade 2) includes four natural sites and all five cultivars. Only one of the natural sites was assessed and was found to be diploid. Assessment of the cultivars revealed that four are tetraploid.

### 3.3 Phylogenetic relationships based on cpDNA

**Figure 4:**
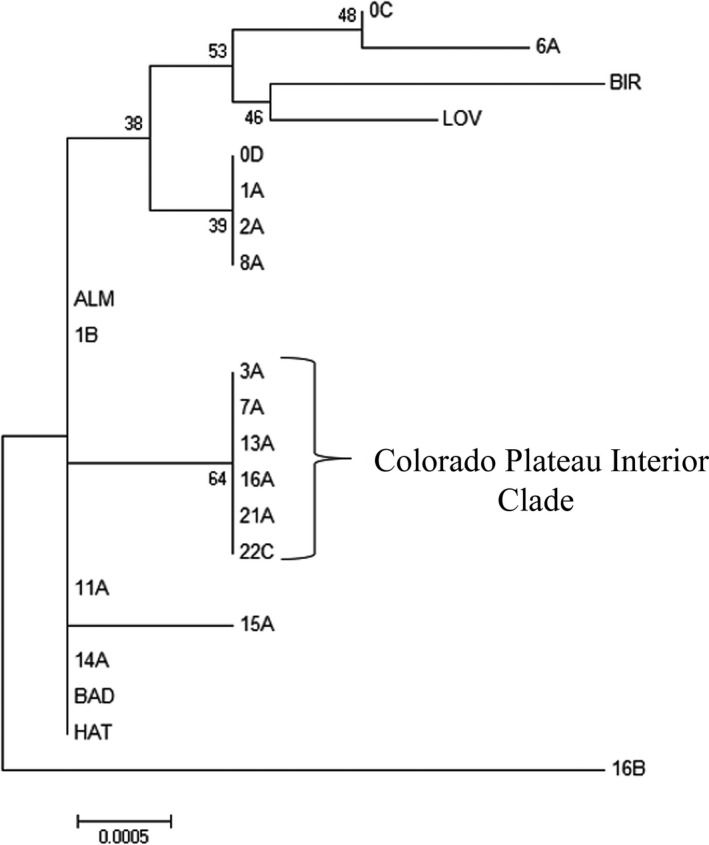
Neighbor‐joining phylogenetic tree as indicated by cpDNA data. The evolutionary distances were computed using the maximum composite likelihood method and are in the units of the number of base substitutions per site. The bootstrap test values (1,000 iterations) are shown next to the branches and did not substantially vary between the neighbor‐joining, maximum parsimony, or maximum‐likelihood methods. The “Interior Colorado Plateau” clade was the only clade consistently supported by the phylogenetic analyses and supports the relationship of “Interior Colorado Plateau” sites identified in the analysis of the amplified fragment length polymorphism (AFLP) data (Figure 3)

Markers psbA‐trnH and 3′trnG–5′trnG2G yielded sequences of 705 and 810 bp in length, respectively. Twenty‐one variable sites in total were detected, four of which were parsimony informative. All three optimality criteria (neighbor‐joining, maximum‐likelihood, and maximum parsimony) produced similar relationships and bootstrap values. Thus, only the neighbor‐joining tree is presented here (Figure 4). Only one clade yielded consistent, moderate bootstrap values (average of 65) including six sample sites (3A, 7A, 13A, 16A, 21A, and 22C) that occupy a linear corridor from just southeast of the boundary, through the interior of the Colorado Plateau toward the northwest to central Utah. All sites identified by the cpDNA analysis as part of the aforementioned “interior sites” are also consistent with Clade 1B as identified by the AFLP‐based phylogeny. Again, this clade is notably composed of tetraploid or mixed ploidy sites.

The cpDNA phylogeny suggests a weak relationship (average bootstrap value of 53) between two natural sampling sites on the far northern and western edge of the Colorado Plateau (0C, 6A) and two cultivars: one from the north (Bird's Eye sourced from Wyoming) and one from the south (Lovington sourced from New Mexico). Two of the four populations have cytotype identified, with one containing only diploid individuals (0C) and the other containing only tetraploid individuals (LOV).

### 3.4 Genetic and environmental correlations

**Figure 5:**
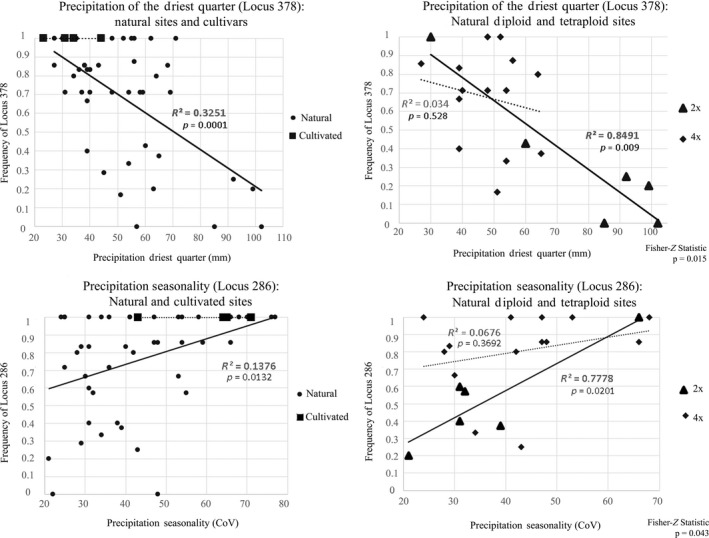
Regression analyses plots of outlier loci and key environmental variables (comparison of natural sites and cultivars). Above plots depict select linear regression analyses of outlier loci and key environmental variable gradients. The graphs on the left show the correlation of locus frequency and environmental variability of natural sites and cultivars. The graphs on the right are paired with those on the left, but show natural sites partitioned by cytotype. Solid lines represent significant correlations while dashed lines are nonsignificant. *p*‐values for the regression analysis are shown directly below the *R*‐squared values while the Fisher‐*Z* correlation comparison *p*‐values for ploidy regressions are shown in the bottom right‐hand corners

Six loci (described above) were identified by BayeScan as outliers and showed significant correlations with one or more of the six nonredundant environmental variables (Table 3, Figure 5, Supporting Information Table S1). Genetic correlation was most strongly associated with MAT, MAP, PDQ, and precipitation of wettest quarter (PWQ). While only loci 378 and 407 demonstrated a correlation between frequency and geographic distance (0.17 and 0.35, respectively), geographic distance was shown to be marginally influential in the regression of frequency and climatic variability. At most, the difference in the *r*‐value between the Mantel and partial‐Mantel tests was 0.04, as was the case with the regression of locus 378 and MAT (0.25 vs. 0.21).

With respect to linear regressions, cultivation, population, and cytotype were all influential in shaping the genetic response to environmental variation. Across all six environmental variables included, the genetic variability of natural populations was significantly correlated with at least one outlier locus per environmental variable, though moderately so with *R*‐squared values ranging from 0.09 to 0.33 (Supporting Information Table S1, Figure 5). While divergent trends between the Colorado Plateau and the off‐Plateau natural populations were defined by the AFLP data as a whole, only precipitation of the coldest quarter at marker 407 was significantly different between the on (*p* = 0.026) and off (*p* = 0.009) Plateau populations, with *R*‐squared values of 0.13 and 0.95, respectively (Fisher‐Z independent correlations test, *p* = 0.009).

All cultivars were originally sourced from off the Colorado Plateau and genetically grouped with the natural off‐Plateau sampling sites (Figures 2 and 3). However, unlike the natural populations, no significant correlation between outlier loci and any of the environmental variables was evident among the cultivars (Figure 5, Supporting Information Table S1). These results suggest that the cultivars may be adapted to the agricultural environment in which they have been cultivated. Alternatively, cultivars may have lost adaptive trait variation through intense selection during the cultivation process (Knapp & Rice, 1994; Schröder & Prasse, 2013a, 2013b).

The different cytotypes demonstrated the most pronounced variance in response to environment (Figure 5, Supporting Information Table S1). Tetraploids were found to occupy climates that were, on average, 50% warmer (MAT of 9°C and 6°C, respectively; *p* = 0.066) and nearly 40% drier than their diploid counterparts (PDQ of 48 and 78 mm, respectively; *p* = 0.023). These climatic differences likely drive adaptive divergence between the ploidy races, which is suggested in five genetic × environmental correlations: locus 378 × MAT, locus 436 × MAT, locus 286 × PS, locus 378 × PDQ, and locus 407 × precipitation of the coldest quarter (PCQ). In all cases, loci from diploid cytotypes were highly and significantly (0.004 < *p *< 0.020) correlated to the environmental variable with an adjusted *R*‐squared value between 0.72 and 0.89. Conversely, tetraploid cytotypes were not significantly (0.369 > *p *> 0.946) correlated to any of the aforementioned environmental variables. The diploid and tetraploid correlations described above were significantly independent from one another (0.001 < *p *< 0.043). Across all loci and all six environmental variables, diploids demonstrated the strongest genetic correlations to environmental variables than any other population groupings, with an average significant *R*‐squared value twice that of tetraploids and nearly fivefold that of natural populations assessed collectively (0.78, 0.37, and 0.17, respectively).

## 4 DISCUSSION

### 4.1 Genetic variation and structure in* B. gracilis*

As hypothesized [1], genetic variation and structure are evident across the sampled region of *B. gracilis’* range. Above‐average expected heterozygosity occurs both on and off the Colorado Plateau, primarily in the central region, while below‐average expected heterozygosity is predominant on the north, east, and western boundary. While the level of within‐population diversity is quite high (70%), it is consistent with patterns of genetic diversity observed in other dominant marker studies of natural *B. gracilis* populations (Aguado‐Santacruz et al., 2004; Phan et al., 2004). While these studies demonstrated higher variation among individuals within populations (88% and 98%, respectively), both studies covered relatively small sample areas, and used different primer combinations, which may account for the higher within‐population variation observed in these studies.

The AFLP data clearly define two distinct groups: one on and the other off the Colorado Plateau. Extensive gene flow throughout the species’ range is supported by this finding, with a distinct interruption of gene flow at the boundary, presumably in large part due to the elevational and geographic barriers presented by the Plateau itself. Although tillering is common, gene flow in *B. gracilis* is also facilitated through seed and pollen dispersal (Aguilera & Lauenroth, 1993; Shaw & Cooper, 1973). Pollen has been shown to have a short window of viability, with dispersal up to 1,800 m within 30 min (Copeland & Hardin, 1970), and seed dispersal through physical attachment to ungulates has been found to be critical to the dispersal of the related species, *Bouteloua curtipendula* (Poaceae) (Laughlin, 2003). These studies, in addition to the study presented here, suggest that gene flow via long‐distance pollen dispersal can greatly contribute to consistent gene flow across continuous habitat.

While genetic structure is observed in populations on and off the Colorado Plateau, the AFLP analysis indicated additional substructure, implying a general divergence of sample sites occupying the south boundary/interior of the Colorado Plateau and sample sites occupying the north/east/west boundary of the Colorado Plateau (Figure 3). The divergence of the boundary and interior sites across the Plateau was mirrored in the resulting cpDNA phylogenies, where an interior Colorado Plateau clade was identified with moderate bootstrap support (Figure 4). Further strengthening the distinction between the boundary and the interior of the Colorado Plateau is the distribution of cytotypes (Table 1), with diploids predominant to the southeast of the Plateau and along the boundary region and tetraploids more common in the interior of the Plateau. We recognize that assessments of genetic diversity and structure typically require larger per‐population sample sizes than those included in the current study. However, our estimates encompass a large number of populations (44 sites and 360 individuals) distributed across the Colorado Plateau. When combined with the AFLP marker loci (94), this constitutes a large number of genotypes (360 × 94 = 33,840), providing a reasonable first estimate of genetic diversity and structure in blue grama.

While distinct climate patterns, such as divergence in MAP (National Research Council, 2007; PRISM Climate Group, 2004) and MAT (PRISM Climate group, 2004), drive the differentiation of ecosystems on the Colorado Plateau, with drylands dominant in the interior and pinyon‐juniper and montane forests dominant at and above the boundary (National Park Service, 2016), other biotic and abiotic factors may further shape the genetic variation observed in blue grama between these two regions. For example, the interior and the boundary regions of the Colorado Plateau also have markedly different topologies, soils, fire return intervals, and drought intensity patterns (Fule, Covington, & Moore, 1997; Miller & Tausch, 2001; Reynolds, Belnap, Reheis, Lamothe, & Luiszer, 2001; The National Drought Mitigation Center, 2017; Webb, Griffiths, & Rudd, 2007).

In addition to biotic and abiotic variables, migration history is also important to take into consideration when evaluating factors that may influence the genetic structure and variability of a species. In a study that assessed the distributions of dominant grasses across the American Midcontinent Plains using several migration models, a blue grama refugia is thought to have occurred in the northeastern corner of New Mexico, just east of the Colorado Plateau on the opposite side of the Southern Rocky Mountain Range (Brown & Gersmehl, 1985). Modeling of the species distribution predicted an outward radiation from this location in an elongated elliptical shape running north to south. The model only assessed gene flow across the Midcontinental Plain, which does not include the Colorado Plateau but does include areas immediately adjacent to it. The ability of the species to rapidly colonize the Plateau moving from the refugia from the west or northwest would have been possible, but may have been slowed due to the fragmented habitat and geographic barriers presented by the Rocky Mountain Range. The model shows the radiation of the species aligning well with the lowest, most gradual transition zone of the Plateau along the southeast boundary in New Mexico. Rapid colonization from this direction, though certainly not the only colonization event possible, is suggested by the genetic structure identified in this study.

Colonization history and isolation by distance (IBD) may also play a role in determining how genetic variation is shaped and ultimately the detection of loci that may be under divergent selection (Lotterhos & Whitlock, 2014). Although our study did not directly test for the influence of these factors, we recognize the importance of doing so, especially within the framework of a more exhaustive sampling design that captures the maximum amount of environmental variation across a species distribution, while minimizing the potential effects of IBD and colonization history. Incorporating this approach can be challenging (Nadeau, Meirmans, Aitken, Ritland, & Isabel, 2016), but may yield additional insight into how environmental variation shapes genetic variation in widespread species, including grasses (e.g., Gray et al., 2014). Given that blue grama occupies a range well beyond our study area, future investigation of genetic × environmental correlations would benefit from more extensive sampling and assessment of divergent loci using recently developed programs that can take into account demographic history (e.g., Bayenv2; Günther & Coop, 2013).

### 4.2 Correlation with environmental variables

As expected [2], *B. gracilis* demonstrates significant genetic covariation with climate, particularly MAT and PDQ (Table 3, Figure 5, Supporting Information Table S1). In addressing question [3], we also found evidence that genetic structure and variability covary with cytotype. While the total genetic divergence between diploids and tetraploids was less than the overall divergence between populations on and off the Colorado Plateau, the influence of cytotype was considerable when assessed in terms of the genetic response to climate (Figure 5), suggesting that genetic variation may be linked to functional trait differentiation. The AFLP data suggest that diploids (prevalent along the boundary) are more sensitive to climate gradients than are tetraploids (prevalent within the interior of the Colorado Plateau) and typically occupy cooler regions that receive more precipitation.

The occupation of blue grama diploids and tetraploids in distinct environments suggests an adaptive advantage of tetraploids across the interior of the Colorado Plateau, where the climate is markedly harsher. In a study by Manzaneda et al. (2011), cytotypes of *Brachypodium distachyon* (Poaceae) were distributed along an aridity gradient and tetraploids were found to have greater water use efficiency than diploids under water‐restricted growing conditions. In *B. gracilis*, temperature was shown to significantly affect physiologically driven traits, including Net CO_2_ assimilation and Rubisco activity in plants collected from high and low elevation sites in the Rocky Mountains (Pitterman & Sage, 2000). This study suggests local adaptation of these photosynthetic‐related traits, which may be functionally divergent due, in part, to genetic differentiation, and the potential influence of cytotype.

In a similar study, tetraploid populations of *Allium przewalkskianum* (Alliaceae) were suggested to have an evolutionary advantage over their diploid counterparts when it came to colonizing and surviving on the arid Qinghai‐Tibetan Plateau in Central and East Asia (Wu, Cui, Milne, Sun, & Liu, 2010; Xie‐Kui, Ao, Zhang, Chen, & Liu, 2014). Not only were tetraploids more commonplace on the arid plateau, cpDNA analysis suggested that independent diploid chromosome duplication events gave rise to tetraploids across the Plateau at least eight times (Wu et al., 2010). This finding supports the claim that the tetraploids may have an adaptive advantage in more arid climates.

Interestingly, a common garden study of *B. gracilis* did not detect any effect of cytotype on measured functional traits (Butterfield & Wood, 2015). The current study, however, detected substantial differences between diploid and tetraploid populations regarding gene‐environment relationships (Figure 5). This discrepancy may be explained by the location of the common garden used in the 2015 study (Flagstaff, AZ), where the MAT is 7.9°C (potentially favorable to tetraploids) and the PDQ is 63 mm (potentially favorable to diploids). Additionally, a diploid site, several tetraploid sites, and mixed cytotype sites all occur within a 20‐mile radius, lending more evidence to the suggestion that the Flagstaff, AZ location, is particularly neutral in regard to climatic pressures on the cytotypic varieties grown in the garden.

In partial support of our question on the genetic distinctness of cultivated *B. gracilis *[4], we found that cultivars currently available on the market were significantly different than samples derived from sites across the Colorado Plateau. Although, contrary to our prediction, the cultivars strongly reflected genetic patterns present in natural sampling sites located off of the Colorado Plateau (Figures 2 and 3), they did not demonstrate the gene × environment correlation observed in their natural counterparts (Figure 5). The observed lack of correlation may, in part, be explained by the agricultural environment in which the cultivars are grown, whereby selective pressure is relaxed for genes (or marker regions) most responsive to environmental variation. In this case, the cultivation environment might include increased climatic stability, warmer growing conditions, irrigation, and the removal of inter‐ and intraspecific competition. However, we recognize that our sampling of cultivated varieties is not exhaustive and that additional surveys are needed to confirm the patterns of adaptive variation observed here. Furthermore, since natural population analogs of the cultivars were not included in this study, additional research is needed to conclusively determine the extent of cultivation effects on environmental adaptation.

### 4.3 Implications for restoration

Since *B. gracilis* is predominantly used in restoration across the Colorado Plateau, findings from this study suggest that increased awareness of seed transfer zones and associated utility will likely be important for restoration success. For example, the distinction between the boundary and the interior of the Colorado Plateau, which was supported collectively by the AFLP data, the cpDNA data, and the cytotypic distribution of this species, suggests that the boundary and interior populations should be considered different ecotypes, consistent with a hypothesis of adaptation within different ecoregions (Hufford & Mazer, 2003). Cytotypes, due to their boundary/interior pattern, may also play a role in assisting with selection of the right seed for restoration and should be a consideration when delineating these broad Colorado Plateau ecoregions. Ecoregions could be further subdivided by environmental variables that appear to shape genetic variation in this species (particularly MAT, MAP, PDQ, PCQ), to develop seed transfer zones for *B. gracilis* across the Colorado Plateau.

The selection of sample sites for this study was based on species‐specific climate modeling (Figure 1) presented by Doherty et al. (2017), which also suggests that *B. gracilis* may be adequately represented across the entire southwest United States with as few as five climate centers. This is reflected, in part, in the genetic analysis, where genetic variation due to differences among individuals within sampling sites across the Colorado Plateau was fairly high (PhiPT = 0.15). This level of within‐population genetic diversity has been documented in other wind‐pollinated species, even within populations that occupy fragmented habitat (Gray et al., 2014; Llorens et al., 2016). This same model could be bolstered with genetic and cytotypic patterns across environmental gradients to help delineate the most appropriate seed transfer zones.

Unfortunately, matching seed transfer zones is not always a possibility when high‐volume seed is required to restore large‐scale disturbances such as wildfire. In addition to the development of robust seed transfer zones, this study also shows that the agricultural development of ecotypes specific to the Colorado Plateau is warranted, with special emphasis on a boundary and interior variety. Methods to produce “facilitated adaptability” in germ lines can be employed from the crossing of individuals collected from diverse natural populations across broad ecoregions to develop new cultivated seed sources (Burton & Burton, 2002).

While ecotype cultivar development of *B. gracilis* has proven difficult in some regions (Carr & Rea, 2013), multisourced ecotype development of the species has been shown to be successful as reported in studies conducted by Fu et al. (2003) and Phan et al. (2004). Ecotypes that were generated with a greater number of individuals (99 vs. 25) and included more source populations (11 vs. 8) had significantly higher genetic diversity (Phan et al., 2004). Further, multisourced ecotype varieties were found to be more genetically varied than the more commonly used single‐sourced cultivars (Fu et al., 2003). While the ecotypes only underwent direct human‐mediated selection for large seed in the source population (G_0_), subtle though significant shifts in marker frequency in cultivated ecotypes was evident after only a single generation of breeding (G_1_). The shift was most pronounced in the ecotype developed from fewer local sources with the fixation of some polymorphic markers arising within only a single generation (Phan et al., 2004). While overall genetic diversity remained quite high after yet another generation (G_2_), these studies highlight that any population in cultivation, regardless of a multisource origin, is susceptible to the loss of genetic diversity. In this regard, marker identification, as was used in this study and others, could be developed to be an affordable and rapid test for recurrent genetic monitoring of cultivated varieties. If such ill‐effects are recognized early, corrective measures could be more efficiently and effectively applied (Burton & Burton, 2002).

### 4.4 Future directions

Additional common garden experiments would be useful in further investigating trends and resolving uncertainties presented by this study. Specifically, a Colorado Plateau garden at a field site colonized by diploids (boundary) and a field site colonized by tetraploids (interior) could substantiate differences observed between cytotypes. A diploid and tetraploid site at off‐Plateau locations could further bring to light the differences observed in this study between populations on and off the Colorado Plateau. The inclusion of currently available cultivars across sites could test their performance across different environments in direct comparison with native genotypes. Seed viability and germination as well as mortality would be important measurements to include to more fully investigate restoration success of different seed sources. A common garden study of this nature could strengthen efforts to develop seed transfer guidelines as well as assist in population selection for cultivated ecotype development.

*Bouteloua gracilis* plays a crucial and expanding role in the restoration of the Colorado Plateau. This study confirms that this species is genetically variable and structurally distinct on and off the Colorado Plateau and is genetically divergent across this area due to environmental and cytotypic variation. Because genetic, cytotypic, and functional trait data have all been acquired for this species, along with methodologies for spatially delineating this information (Butterfield & Wood, 2015; Doherty et al., 2017), the establishment of well‐defined seed transfer zones is readily attainable. Additionally, seed currently available on the market for this species has been sourced from off the Colorado Plateau and has been demonstrated in this study to exhibit extensive genetic departure from and lack of covariation with environmental variability relative to natural populations on the Plateau. This study underscores the need for the development of new cultivated varieties to provide suitable seed for large‐scale restoration that encompasses the variation observed across diverse environments on the Colorado Plateau.

## CONFLICT OF INTEREST

None declared.

## AUTHOR CONTRIBUTIONS

KT performed all of the molecular genetic experiments and data analysis and wrote the first draft of the manuscript. GJA oversaw the laboratory work and analyses and edited the final version of the manuscript.

## DATA ACCESSIBILITY

The AFLP data generated for this study will be made available for public use on http://data/dryad.org. The regression summary of outlier loci and environmental variables is included as a Supporting Information Table S1.
