# Supplementary material for: Environmental variation shapes genetic variation in Bouteloua gracilis: Implications for restoration management of natural populations and cultivated varieties in the southwestern United States
Source: Ecol Evol. 2018 Dec 26;9(1):482–99. doi: 10.1002/ece3.4767 (PMC6342110; doi:10.1002/ece3.4767)
Supplement: Supplementary file 1 [file ECE3-9-482-s001.docx]

| **Locus** | **Variable** | **Group** | **N** | **R-squared** | **Adjusted R-squared** | **Significance of regression (p-value)** | **Sig.** | **Fisher Z Comparison of Correlations (p-value)** | **Sig.** |
| --- | --- | --- | --- | --- | --- | --- | --- | --- | --- |
| **193** | **Mean Annual Temp** | Natural Sampling Sites | 44 | 0.29 | 0.27 | 0.0002 | *** | 0.130 |  |
|  |  | Cultivated Varieties | 4 | 0.30 | -0.05 | 0.4529 |  |  |  |
|  |  | Colorado Plateau Sites | 39 | 0.22 | 0.20 | 0.0023 | ** | 0.135 |  |
|  |  | Off-Plateau Sites (Natural) | 5 | 0.75 | 0.67 | 0.0579 | ~ |  |  |
|  |  | Natural Sites with Ploidy Data | 26 | 0.16 | 0.13 | 0.0413 | * |  |  |
|  |  | Diploid (2x) | 6 | 0.33 | 0.17 | 0.2294 |  | 0.209 |  |
|  |  | Tetraploid (4x) | 14 | 0.02 | -0.06 | 0.6549 |  |  |  |
| **286** | **Mean Annual Temp** | Natural Sampling Sites | 44 | 0.16 | 0.14 | 0.0068 | ** | 0.338 |  |
|  |  | Cultivated Varieties | 4 | NA | NA | NA |  |  |  |
|  |  | Colorado Plateau Sites | 39 | 0.12 | 0.10 | 0.0309 | * | 0.146 |  |
|  |  | Off-Plateau Sites (Natural) | 5 | 0.66 | 0.54 | 0.0969 | ~ |  |  |
|  |  | Natural Sites with Ploidy Data | 26 | 0.25 | 0.22 | 0.0085 | ** |  |  |
|  |  | Diploid (2x) | 6 | 0.61 | 0.52 | 0.0658 | ~ | 0.068 | ~ |
|  |  | Tetraploid (4x) | 14 | 0.01 | -0.08 | 0.7796 |  |  |  |
| ***378*** | ***Mean Annual Temp*** | Natural Sampling Sites | 44 | 0.24 | 0.22 | 0.0007 | *** | 0.298 |  |
|  |  | Cultivated Varieties | 4 | NA | NA | NA |  |  |  |
|  |  | Colorado Plateau Sites | 39 | 0.17 | 0.15 | 0.0094 | ** | 0.138 |  |
|  |  | Off-Plateau Sites (Natural) | 5 | 0.71 | 0.61 | 0.0738 | ~ |  |  |
|  |  | Natural Sites with Ploidy Data | 26 | 0.32 | 0.29 | 0.0027 | ** |  |  |
|  |  | Diploid (2x) | 6 | 0.89 | 0.87 | 0.0045 | ** | 0.001 | * |
|  |  | Tetraploid (4x) | 14 | 0.04 | -0.04 | 0.4989 |  |  |  |
| **436** | **Mean Annual Temp** | Natural Sampling Sites | 44 | 0.24 (-) | 0.06 (-) | 0.1071 |  | 0.083 |  |
|  |  | Cultivated Varieties | 4 | 0.67 (+) | 0.51 (+) | 0.1779 |  |  |  |
|  |  | Colorado Plateau Sites | 39 | 0.02 | -0.01 | 0.3809 |  | 0.267 |  |
|  |  | Off-Plateau Sites (Natural) | 5 | 0.29 | 0.05 | 0.3531 |  |  |  |
|  |  | Natural Sites with Ploidy Data | 26 | 0.10 | 0.06 | 0.1146 |  |  |  |
|  |  | Diploid (2x) | 6 | 0.80 | 0.75 | 0.0160 | * | 0.013 | * |
|  |  | Tetraploid (4x) | 14 | 0.00 | -0.08 | 0.9464 |  |  |  |
| **471** | **Temp Seasonality** | Natural Sampling Sites | 44 | 0.09 | 0.07 | 0.0487 | * | 0.075 | ~ |
|  |  | Cultivated Varieties | 4 | 0.69 (-) | 0.53 (-) | 0.1713 |  |  |  |
|  |  | Colorado Plateau Sites | 39 | 0.18 | 0.15 | 0.0078 | ** | 0.227 |  |
|  |  | Off-Plateau Sites (Natural) | 5 | 0.01 | -0.32 | 0.8759 |  |  |  |
|  |  | Natural Sites with Ploidy Data | 26 | 0.35 | 0.32 | 0.0016 | ** |  |  |
|  |  | Diploid (2x) | 6 | 0.29 | 0.23 | 0.0955 | ~ | 0.304 |  |
|  |  | Tetraploid (4x) | 14 | 0.54 | 0.43 | 0.0453 | * |  |  |

| **Locus** | **Variable** | **Group** | **N** | **R-squared** | **Adjusted R-squared** | **Significance of regression (p-value)** | **Sig.** | **Fisher Z Comparison of Correlations (p-value)** | **Sig.** |
| --- | --- | --- | --- | --- | --- | --- | --- | --- | --- |
| ***378*** | ***Mean Annual Precip*** | Natural Sampling Sites | 44 | 0.13 | 0.11 | 0.0179 | * | Highly unequal range |  |
|  |  | Cultivated Varieties | 4 | NA | NA | NA |  |  |  |
|  |  | Colorado Plateau Sites | 39 | 0.09 | 0.07 | 0.0639 | ~ | 0.177 |  |
|  |  | Off-Plateau Sites (Natural) | 4 | 0.57 | 0.43 | 0.1408 |  |  |  |
|  |  | Natural Sites with Ploidy Data | 26 | 0.24 | 0.21 | 0.0110 | * |  |  |
|  |  | Diploid (2x) | 6 | 0.56 | 0.44 | 0.0890 | ~ | 0.158 |  |
|  |  | Tetraploid (4x) | 14 | 0.09 | 0.01 | 0.2959 |  |  |  |
| **286** | **Mean Annual Precip** | Natural Sampling Sites | 44 | 0.09 | 0.07 | 0.0505 | * | Highly unequal range |  |
|  |  | Cultivated Varieties | 4 | NA | NA | NA |  |  |  |
|  |  | Colorado Plateau Sites | 39 | 0.06 | 0.04 | 0.1218 |  | 0.187 |  |
|  |  | Off-Plateau Sites (Natural) | 4 | 0.52 | 0.35 | 0.1716 |  |  |  |
|  |  | Natural Sites with Ploidy Data | 26 | 0.30 | 0.27 | 0.0041 | ** |  |  |
|  |  | Diploid (2x) | 6 | 0.37 | 0.21 | 0.2033 |  | 0.398 |  |
|  |  | Tetraploid (4x) | 14 | 0.24 | 0.17 | 0.0768 | ~ |  |  |
| **471** | **Mean Annual Precip** | Natural Sampling Sites | 44 | 0.18 | 0.16 | 0.0045 | ** | Highly unequal range |  |
|  |  | Cultivated Varieties | 4 | 0.06 | -0.41 | 0.7562 |  |  |  |
|  |  | Colorado Plateau Sites | 39 | 0.25 | 0.23 | 0.0013 | ** | 0.356 |  |
|  |  | Off-Plateau Sites (Natural) | 4 | 0.07 | -0.24 | 0.6615 |  |  |  |
|  |  | Natural Sites with Ploidy Data | 26 | 0.35 | 0.32 | 0.0015 | ** |  |  |
|  |  | Diploid (2x) | 6 | 0.15 | -0.06 | 0.4439 |  | 0.33 |  |
|  |  | Tetraploid (4x) | 14 | 0.36 | 0.31 | 0.0223 | * |  |  |
| **286** | **Precip Seasonality** | Natural Sampling Sites | 44 | 0.14 | 0.12 | 0.0132 | * | 0.382 |  |
|  |  | Cultivated Varieties | 4 | NA | NA | NA |  |  |  |
|  |  | Colorado Plateau Sites | 39 | 0.13 | 0.10 | 0.0266 | * | 0.443 |  |
|  |  | Off-Plateau Sites (Natural) | 5 | 0.20 | -0.07 | 0.4561 |  |  |  |
|  |  | Natural Sites with Ploidy Data | 26 | 0.18 | 0.15 | 0.0298 | * |  |  |
|  |  | Diploid (2x) | 6 | 0.78 | 0.72 | 0.0201 | * | 0.043 | * |
|  |  | Tetraploid (4x) | 14 | 0.07 | -0.01 | 0.3692 |  |  |  |
| ***378*** | ***Precip Driest Quarter*** | Natural Sampling Sites | 44 | 0.33 | 0.31 | 0.0001 | *** | Highly unequal range |  |
|  |  | Cultivated Varieties | 4 | NA | NA | NA |  |  |  |
|  |  | Colorado Plateau Sites | 39 | 0.26 | 0.24 | 0.0009 | *** | 0.197 |  |
|  |  | Off-Plateau Sites (Natural) | 5 | 0.69 | 0.58 | 0.0827 | ~ |  |  |
|  |  | Natural Sites with Ploidy Data | 26 | 0.46 | 0.44 | 0.0001 | *** |  |  |
|  |  | Diploid (2x) | 6 | 0.85 | 0.81 | 0.0090 | ** | 0.015 | * |
|  |  | Tetraploid (4x) | 14 | 0.03 | -0.05 | 0.5283 |  |  |  |

| **Locus** | **Variable** | **Group** | **N** | **R-squared** | **Adjusted R-squared** | **Significance of regression (p-value)** | **Sig.** | **Fisher Z Comparison of Correlations (p-value)** | **Sig.** |
| --- | --- | --- | --- | --- | --- | --- | --- | --- | --- |
| ***407*** | ***Precip Coldest Quarter*** | Natural Sampling Sites | 44 | 0.18 | 0.16 | 0.0052 | ** | Highly unequal range |  |
|  |  | Cultivated Varieties | 4 | NA | NA | NA |  |  |  |
|  |  | Colorado Plateau Sites | 39 | 0.13 | 0.10 | 0.0265 | * | 0.009 | ** |
|  |  | Off-Plateau Sites (Natural) | 5 | 0.95 | 0.93 | 0.0048 | ** |  |  |
|  |  | Natural Sites with Ploidy Data | 26 | 0.17 | 0.13 | 0.0374 | * |  |  |
|  |  | Diploid (2x) | 6 | 0.80 | 0.75 | 0.0158 | * | 0.044 | * |
|  |  | Tetraploid (4x) | 14 | 0.11 | 0.03 | 0.2526 |  |  |  |

Supplemental Table A: Regression summary of outlier loci and environmental variables (contd’)

Outlier loci indicated to be nominally influenced by geographic distance (378 and 407) are italicized. The (+) or (-) symbols in the R-squared columns are used only when opposing trends are substantially evident between different groups. Fisher-Z correlation analysis values were generated to ascertain significant difference in correlation values between two groups. The significance, or “Sig.” column is associated with both the linear regression p-values as well as the Fisher-Z comparison of correlations test and is symbolized to bring attention to those findings that are below or are near an alpha value of 0.05. The (~) symbol identifies a p-value of between 0.05 and 0.1, the (*) symbol represents p-values that are below 0.05, the (**) symbol represents p-values that are below 0.01, and the (***) symbol represents p-values that are below 0.001.
